# The non-linear link between remnant cholesterol and diabetic retinopathy: a cross-sectional study in patients with type 2 diabetic mellitus

**DOI:** 10.1186/s12902-022-01239-5

**Published:** 2022-12-21

**Authors:** Wushan Pan, Yong Han, Haofei Hu, Yongcheng He

**Affiliations:** 1 Department of Nephrology, Kaifeng Central Hospital, Kaifeng, 475000 Henan Province China; 2 Department of Emergency, Shenzhen Second People’s Hospital, Guangdong Province, Shenzhen, 518000 China; 3 Department of Emergency, The First Affiliated Hospital of Shenzhen University, Guangdong Province, Shenzhen, 518000 China; 4 Department of Nephrology, Shenzhen Second People’s Hospital, Guangdong Province, Shenzhen, 518000 China; 5 Department of Nephrology, The First Affiliated Hospital of Shenzhen University, Guangdong Province, Shenzhen, 518000 China; 6 Department of Nephrology, Shenzhen Hengsheng Hospital, No.3002 Sungang Road, Futian District, Shenzhen, 518000 Guangdong Province China

**Keywords:** Diabetic retinopathy, Proliferative diabetic retinopathy, Remnant cholesterol, Cross-sectional study, Non-linear, Generalized additive model

## Abstract

**Objective:**

Research on residual cholesterol (RC) and diabetic retinopathy (DR) remains limited. As a result, the current study was designed to investigate the relationship between RC and DR in patients with type 2 diabetic mellitus (T2DM).

**Methods:**

This cross-sectional study consecutively and non-selectively collected a total of 1964 type 2 diabetic mellitus patients in two hospitals in Taiwan from April 2002 to November 2004. A binary logistic regression model was then used to assess the independent relationship between RC level and DR and proliferative diabetic retinopathy (PDR). A generalized additive model (GAM) and smooth curve fitting were used to investigate the actual shape of the curve between them. It was stated that the data had been uploaded to the website:https://journals.plos.org/plosone.

**Results:**

The average age of the participants was 64.10+/− 11.32 years old, with 42.92% being male. The prevalence of DR and PDR was 35.13 and 18.13%, respectively. The mean RC level was 30.57 ± 14.60 mg/dL. We found no significant association between RC and DR (OR = 1.001; 95% CI 0.991, 1.011) or PDR (OR = 1.008; 95% CI 0.995, 1.021) based on a fully adjusted logistic regression model. Results remained robust across a series of sensitivity analyses. However, a non-linear relationship was detected between RC and DR. Using a two-piece logistic regression model and a recursive algorithm, we found an inflection point of RC was 13.0 mg/dL. A 1-unit increase in the RC level was associated with 19.4% greater adjusted odds of DR (OR = 1.194; 95% CI 1.070, 1.333) when RC < 13.0 mg/dL. There was also a non-linear relationship between RC and PDR, and the inflection point of the RC was 39.0 mg/dL. When RC < 39.0 mg/dL, a 1-unit increase in the RC level was associated with 2.1% greater adjusted odds of PDR (OR = 1.021; 95% CI 1.004, 1.038).

**Conclusion:**

This study demonstrates a non-linear relationship between RC and DR or PDR in type 2 diabetic mellitus patients. Our findings provide new insights into advancing research on the link between RC and DR or PDR.

**Supplementary Information:**

The online version contains supplementary material available at 10.1186/s12902-022-01239-5.

## Background

The metabolic syndrome has been linked to retinopathy and diabetic retinopathy (DR) potentiation [1]. Lipids are risk factors for macrovascular disease. According to some recent studies, conventional serum lipid levels such as total cholesterol (TC), triglyceride (TG), low-density lipoprotein cholesterol (LDL-c), and high-density lipoprotein cholesterol (HDL-c) were not significantly associated with DR [2, 3]. Remnant cholesterol (RC) is the cholesterol content of triglyceride-rich lipoproteins, which are very-low-density lipoprotein (VLDL) and intermediate-density lipoprotein (IDL) in the fasting state and the non-fasting state chylomicron remnants [4]. Previous research has shown that RC is the primary factor mediating the residual risk of cardiovascular events, and that it is also independently associated with atherosclerosis [5–7]. Researchers have begun investigating the link between RC and diabetic retinopathy in recent years. A Chinese cross-sectional study including 456 patients showed that a high RC level is associated with DR in type 2 diabetes mellitus (T2DM) [2]. Another study found that RC could predict diabetic nephropathy and retinopathy progression in type 1 diabetes [8]. However, research on the relationship between RC and DR in type 2 diabetes patients is still limited. No studies have simultaneously explored RC’s association with DR and proliferative diabetic retinopathy (PDR) in T2DM patients. In addition, the existence of a linear or non-linear relationship between RC and DR still needs to be further explored. Therefore, we conducted a cross-sectional study investigating the relationship between RC and diabetic retinopathy in T2DM patients.

## Methods

### Study design

This study used a cross-sectional design. The target-independent variable was RC. The dependent variable was diabetic retinopathy (dichotomous variable: 1 = DR, 0 = non-DR) and proliferative diabetic retinopathy (dichotomous variable: 1 = PDR, 0 = NPDR).

### Data source

We downloaded the raw data freely from (https://journals.plos.org/plosone), provided by Chen et al.. From: Abnormally Low or High Ankle-Brachial Index Is Associated with Proliferative Diabetic Retinopathy in Type 2 Diabetic Mellitus Patients. 10.1371/journal.pone.0134718. This is an open-access article published in accordance with the Creative Commons Attribution License, which enables unlimited use, distribution, and reproduction in any form, as long as the original author and source are acknowledged [9]. Here, we would like to thank the authors for providing the data.

### Study population

All patients with T2DM who visited the diabetic clinic in the Internal Medicine outpatient departments of two hospitals in southern Taiwan between April 2002 and November 2004 were included in the study. The original researchers gathered the consecutive cases in a non-selective manner. To ensure participants’ privacy, the original researchers encoded their identity information with non-traceable codes. The data came from the hospital’s electronic medical record system, including physical medical, medication, laboratory, and imaging data. This research was conducted under the approval of the institutional review board of the Kaohsiung Medical University Hospital (No. KMUHIRB-E-20150029). All participants have given informed consent to take part in the study. All methods were performed in accordance with the relevant guidelines and regulations by including a statement in the Declarations section [9].

**Fig. 1:**
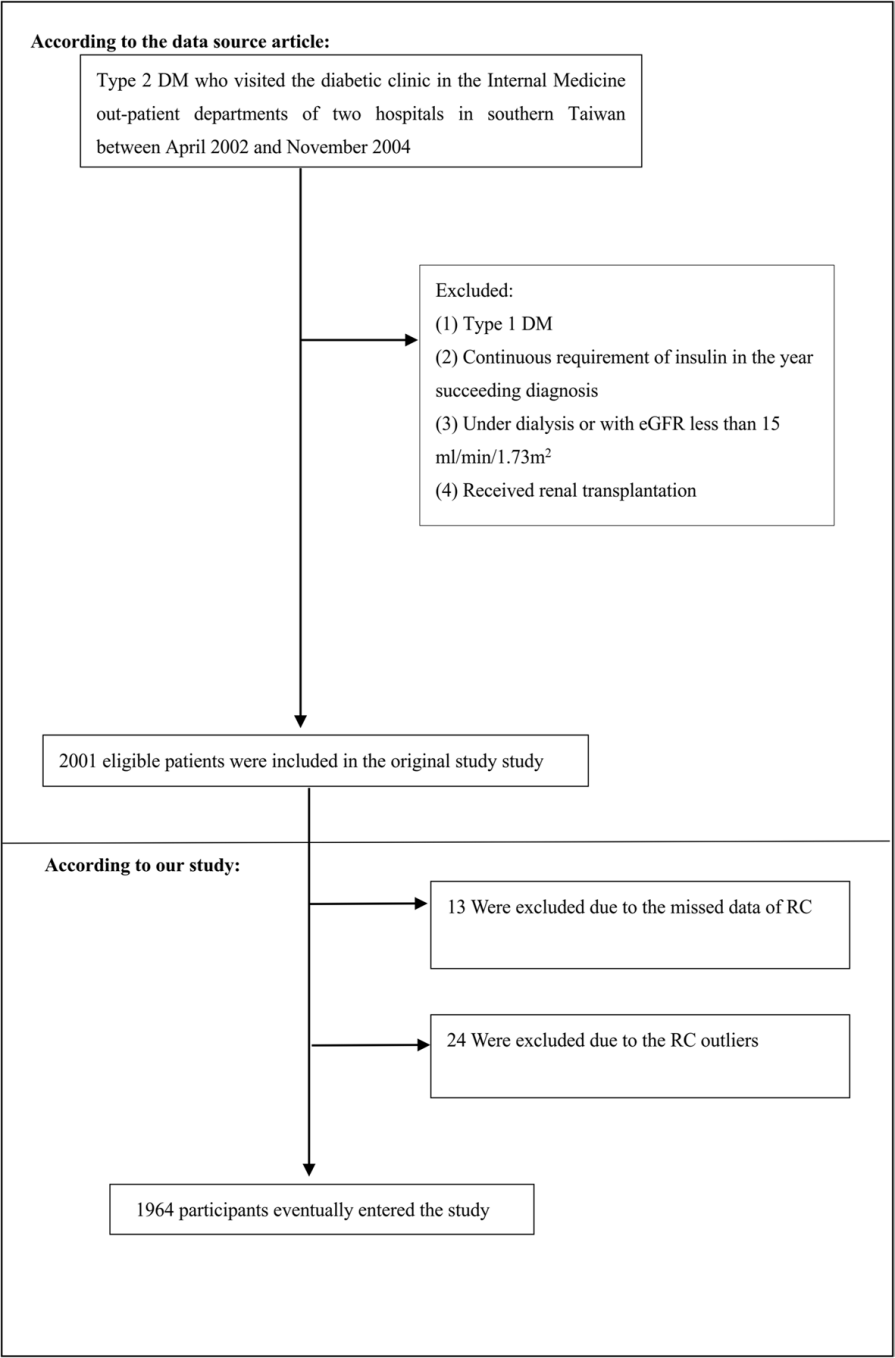
Flowchart of study participants. Figure 1 showed the inclusion of patients. 2001 patients were assessed for eligibility in the original study. We excluded patients with missing values of RC (*n* = 13) and RC (*n* = 24) outliers. The final analysis included 1964 subjects in the present study

Individuals were excluded from the original study if they met any of the following criteria [9]: (1) patients with type 1 DM; (2) patients with estimated glomerular filtration rate (eGFR) < 15 ml/min/1.73m^2^ or under dialysis; (3) patients who received a kidney transplant. Finally, 2001 patients were involved in the original research. Our research further excluded participants with missing values of RC. To reduce interference, we excluded outliers in RC, which were not included in the range of the means ± three standard deviations (SD) [10]. The final analysis included 1964 subjects (843 men and 1121 women) in the present study (see the flowchart for details in Fig. 1).

### Variables

#### Remnant cholesterol

The information on RC was recorded as a continuous variable. The detailed process of measuring RC was described as follows: RC was calculated as non-high-density lipoprotein cholesterol (non-HDL-c) – LDL-c [11], where non-HDL-c = TC – HDL-c [12].

#### Diabetic Retinopathy

Our interesting dependent variable was diabetic retinopathy (dichotomous variable: 1 = DR, 0 = non-DR) and proliferative diabetic retinopathy (dichotomous variable: 1 = PDR, 0 = non-PDR). DR was assessed by experienced ophthalmologists while the patient’s pupils were dilated. Fluorescein angiography was performed if necessary. DR was classified as non-DR, NPDR, and PDR stages [13].

#### Covariates

The covariates involved in this study were selected based on the original study, our clinical experience, and other studies on risk factors for DR. Based on the above principles; thus, the following variables were used as covariates: (1) continuous variables: body mass index (BMI), age, diastolic blood pressure (DBP), eGFR, fasting plasma glucose (FPG), systolic blood pressure (SBP), hemoglobin A1c (HbA1c), and TG; (2) categorical variables: sex, history of coronary artery disease and cerebrovascular disease, angiotensin-converting enzyme inhibitor (ACEI) and/or angiotensin II receptor blocker (ARB) use, β-blocker use, diuretic use, calcium channel blocker (CCB) use, and microalbuminuria.

The BMI was calculated as the ratio of weight divided by the square of height. An autoanalyzer measured laboratory data from fasting blood samples [14]. eGFR was calculated using the 4-variable equation in the Modification of Diet in Renal Disease (MDRD) study [15]. Urine albumin and creatinine were measured on a spot urine sample. Microalbuminuria was defined as the ratio of urine albumin to creatinine of ≥30 mg/gm [9].

### Statistical analysis

We stratified the participants by quartiles of the RC level. Characteristics of patients were expressed as mean (standard deviation) (normal distribution) or median (range) (non-normal distribution) for continuous variables, and as percentages for categorical variables. We used the One-Way ANOVA test (normal distribution), χ2 (categorical variables), or Kruskal-Whallis H test (skewed distribution) to test for differences among different RC groups.

To explore the link between RC and DR or PDR, we constructed three distinct models using a logistic regression model, including a non-adjusted model (Crude model: no covariates were adjusted), minimally-adjusted model (Model I: only sociodemographic variables and treatment situation were adjusted, including age, SBP, sex, DBP, BMI, history of coronary artery disease and cerebrovascular disease, β-blocker use, ACEI and/or ARB use, diuretic use, and CCB use) and fully-adjusted model (covariates presented in Table 1 were adjusted, including age, SBP, sex, DBP, BMI, history of coronary artery disease and cerebrovascular disease, β-blocker use, ACEI and/or ARB use, diuretic use, CCB use, FPG, HbA1c, eGFR, TG, and microalbuminuria). Effect sizes (OR) with 95% confidence intervals (CI) were recorded. We adjusted them when the covariances were added to the model and the OR changed by 10% or greater [16].

**Table 1:** The baseline characteristics of participants

RC Quartile	Q1(< 20.0)	Q2(20.0-29.0)	Q3(29.0-38.0)	Q4(≥38.0)	*P*-value
Participants	438	533	485	508	
Age (year)	63.5 ± 12.2	64.5 ± 11.0	64.5 ± 11.6	63.9 ± 10.6	0.487
BMI (kg/m^2^)	24.7 ± 3.2	25.9 ± 3.7	26.0 ± 3.7	26.5 ± 3.3	< 0.001
SBP (mmHg)	133.8 ± 19.3	133.2 ± 17.3	135.4 ± 18.8	137.0 ± 19.5	0.005
DBP (mmHg)	76.6 ± 10.8	76.6 ± 10.7	78.1 ± 11.0	79.7 ± 12.3	< 0.001
PP (mmHg)	57.2 ± 15.9	56.7 ± 15.0	57.4 ± 15.2	57.3 ± 15.1	0.861
HbA1c(%)	7.4 ± 1.5	7.5 ± 1.6	7.6 ± 1.6	8.0 ± 1.7	< 0.001
FPG (mg/dL)	139.4 ± 49.4	144.0 ± 51.9	146.7 ± 44.6	161.1 ± 54.3	< 0.001
TC (mg/dL)	162.9 ± 28.1	174.8 ± 28.2	186.2 ± 28.6	211.2 ± 37.7	< 0.001
TG (mg/dL)	86.4 ± 30.0	113.9 ± 36.7	145.1 ± 48.3	233.5 ± 112.5	< 0.001
LDL-c (mg/dL)	93.9 ± 24.7	100.7 ± 24.6	105.0 ± 24.1	116.2 ± 31.1	< 0.001
HDL-c (mg/dL)	54.8 ± 14.0	50.3 ± 12.8	48.7 ± 12.3	45.2 ± 11.5	< 0.001
non-HDL (mg/dL)	108.0 ± 25.2	124.6 ± 24.8	137.6 ± 24.5	166.0 ± 33.2	< 0.001
RC (mg/dL)	14.2 ± 3.9	23.9 ± 2.5	32.6 ± 2.5	49.8 ± 12.0	< 0.001
Scr (mg/dL)	1.0 ± 0.3	1.1 ± 0.3	1.1 ± 0.4	1.1 ± 0.4	0.074
eGFR (mL/min/1.73 m^2^)	71.1 ± 18.6	68.7 ± 18.9	68.2 ± 20.3	67.0 ± 20.6	0.016
Gender					0.169
Female	234 (53.4%)	302 (56.7%)	277 (57.1%)	308 (60.6%)	
Male	204 (46.6%)	231 (43.3%)	208 (42.9%)	200 (39.4%)	
Microalbuminuria (%)	115 (26.3%)	165 (31.0%)	161 (33.2%)	242 (47.6%)	< 0.001
Coronary artery disease (%)	73 (16.7%)	84 (15.8%)	76 (15.7%)	95 (18.7%)	0.538
Cerebrovascular disease (%)	13 (3.0%)	30 (5.6%)	18 (3.7%)	35 (6.9%)	0.019
β-blocker use (%)	74 (16.9%)	123 (23.1%)	124 (25.6%)	137 (27.0%)	0.002
ACEI and/or ARB use (%)	299 (68.3%)	394 (73.9%)	364 (75.1%)	389 (76.6%)	0.026
Diuretic use (%)	173 (39.5%)	246 (46.2%)	234 (48.2%)	251 (49.4%)	0.013
Calcium channel blocker use (%)	136 (31.1%)	192 (36.0%)	211 (43.5%)	230 (45.3%)	< 0.001

Values are *n* (%) or mean ± SD

*Abbreviations:* *HbA1c* Hemoglobin A1c, *eGFR* Estimated glomerular filtration rate, *BMI* Body mass index, *Scr* Serum creatinine, *FPG* Fasting plasma glucose, *HDL-c* High-density lipoprotein cholesterol, *LDL-c* Low-density lipoprotein cholesterol, *TC* Total cholesterol, *TG* Triglyceride, *SBP* Systolic blood pressure, *DBP* Diastolic blood pressure, *PP* Pulse pressure, *non-HDL* Non-high-density lipoprotein cholesterol, *RC* Remnant cholesterol, *ACEI* Angiotensin-converting enzyme inhibitor, *ARB* Angiotensin II receptor blocker

Since methods based on binary logistic regression models were often suspected of being unable to handle non-linear models, therefore, non-linearity between RC and diabetic retinopathy was addressed using the Generalized additive model and the smooth curve fitting (penalized spline method). If a non-linearity was found, we first calculated the inflection point using a recursive algorithm. We then built a two-piece logistic regression model on either side of the inflection point. The best-fitting model was determined based on the *p*-value of the log of the likelihood ratio [17].

The number of participants with missing data of HbA1c, FPG, Scr, eGFR, β-blocker use, ACEI and/or ARB use, diuretic use, and CCB use was 4(0.20%), 2(0.10%), 4(0.20%), 4(0.20%), 7(0.36%), 7(0.36%),7(0.36%), and 7(0.36%), respectively. Multiple imputations were used to handle the missing data of covariants [18]. The imputation model included age, SBP, BMI, DBP, sex, coronary artery disease, cerebrovascular disease, β-blocker use, ACEI and/or ARB use, diuretic use, CCB use, FPG, HbA1c, Scr, eGFR, TG, and microalbuminuria. Missing data analysis procedures use missing-at-random (MAR) assumptions [19].

To test the robustness of our results, we performed a series of sensitivity analyses. According to the quartile, we converted RC into a categorical variable. We calculated the P for the trend to verify the results for RC as the continuous variable and to examine the possibility of non-linearity. Besides, due to the limitation of the generalized linear model in addressing non-linearity, we performed a GAM model to adjust for covariates in model III [17]. Additionally, we explored the potential for unmeasured confounding between RC and the risk of DR by calculating E-values [20].

All the analyses were performed with the statistical software packages R (http://www.R-project.org, The R Foundation) and EmpowerStats (http://www. empowerstats.com, X&Y Solutions, Inc., Boston, MA). *P* values less than 0.05 (two-sided) were considered statistically significant.

## Results

### Characteristics of participants

Table 1 provided the demographic and clinical characteristics of participants included in the study. The final analysis included 1964 participants, 42.92% of whom were male and had a mean age of 64.10 11.32 years. The prevalence of DR and PDR was 35.13 and 18.13%, respectively. And the mean RC was 30.57 ± 14.60 mg/dL. No statistical difference was found in the characteristics in terms of age, pulse pressure (PP), Scr, and coronary artery disease in different groups of RC (quartile) (all *P* values > 0.05). When we set the Q1 (RC < 20.0 mg/dL) group as a reference, the higher value or proportion of SBP, BMI, DBP, FPG, LDL-c, HbA1c, TC, TG, non-HDL, RC, males, microalbuminuria, cerebrovascular disease, β-blocker use, ACEI and/or ARB use, diuretic use, and CCB use were observed were detected in the Q4 (RC ≥ 38.0 mg/dL) group, while the lower level of eGFR and HDL-c were observed in the Q4 group.

**Fig. 2:**
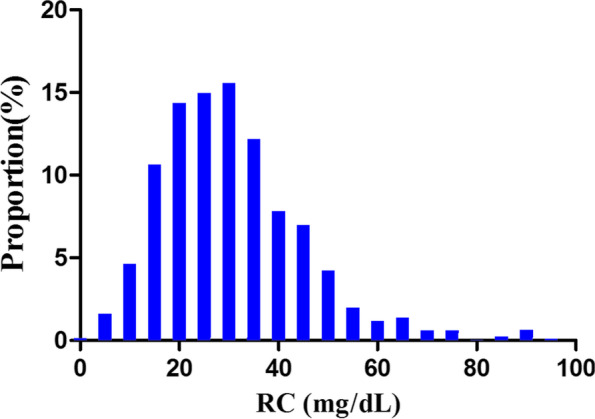
Distribution of RC. Figure 2. It presented a normal distribution of RC in the range from 1 to 93 mg/dL, with a mean of 30.57 mg/dL

Figure 2 showed the distribution of RC levels. It presented a normal distribution from 1.0 to 93.0 mg/dL, with a mean value of 30.57 mg/dL. Patients were divided into three groups based on their diabetic retinopathy status (non-DR, nonPDR, and PDR) (Table S1). The RC levels in the three groups were shown in Fig. S1. The results showed that there were no statistically significant differences in the distribution levels of RC in the three groups (*P* = 0.6665).

### The prevalence rate of DR and PDR

Table S2 revealed that a total of 690 participants had DR. The total prevalence rate of all participants was 35.13% (33.02-37.25%). Specifically, the prevalence rates of the four RC groups were 30.37% (26.04-34.69%), 38.27% (34.13-42.41%), 36.49% (32.20-40.79%), and 34.65% (30.49-38.80%), respectively.

Table S2 also revealed that 356 participants had PDR in total. The total prevalence rate of all participants was 18.13% (16.42-19.83%). Specifically, the prevalence rates of the four RC groups were 14.84% (11.50-18.18%), 17.45% (14.22-20.68%), 20.00% (16.43-23.57%), and 19.88% (16.40-23.36%), respectively.

### The results of univariate analyses using the binary logistic regression model

The univariate analyses showed DR had nothing to do with gender, DBP, BMI, TC, FPG, TG, LDL-c, RC, non-HDL-c, β-blocker use (all *P* > 0.05), but positively related to age, HbA1c, SBP, Scr, PP, microalbuminuria, history of coronary artery disease and cerebrovascular disease, ACEI and/or ARB use, diuretic use, CCB use, and negatively related to HDL-c, eGFR (all *P* < 0.05; Table S3).

The results also showed that age, gender, BMI, DBP, FPG, TC, TG, LDL-c, HDL-c, non-HDL-c, RC, coronary artery disease, and β-blocker use were not related to PDR (All *P*-values> 0.05), but SBP, PP, HbA1c, Scr, microalbuminuria, cerebrovascular disease, ACEI and/or ARB use, diuretic use, and calcium channel blocker use were positively associated with PDR, and eGFR were negatively connected with PDR (all P < 0.05; Table S3).

### The results of multivariate analyses using the binary logistic regression model

We found no significant association between RC and DR in the crude model, model I, and model II (Table 2). The trend of ORs and 95% CI were robust irrespective of the type of covariates adjusted (Crude model: OR = 1.001; 95% CI: 0.995-1.008, the model I: OR = 1.000; 95% CI: 0.993-1.006, model II: OR = 1.001; 95% CI: 0.991-1.011).

**Table 2:** Relationship between RC and DR or PDR in different models

Variable	Crude model (OR,95%CI, P)	Model I (OR,95%CI, P)	Model II (OR,95%CI, P)	Model III (OR,95%CI, P)
DR				
RC (mg/dL)	1.001 (0.995, 1.008) 0.69304	1.000 (0.993, 1.006) 0.88369	1.001 (0.991, 1.011) 0.85549	0.999 (0.989, 1.010) 0.89030
RC (Quartile)				
Q1	Ref.	Ref.	Ref.	Ref.
Q2	1.422 (1.087, 1.860) 0.01012	1.405 (1.067, 1.850) 0.01547	1.397 (1.055, 1.850) 0.01949	1.314 (0.980, 1.762) 0.06836
Q3	1.318 (1.001, 1.735) 0.04921	1.263 (0.951, 1.676) 0.10622	1.282 (0.952, 1.727) 0.10155	1.184 (0.855, 1.639) 0.30996
Q4	1.216 (0.925, 1.598) 0.16182	1.144 (0.860, 1.520) 0.35563	1.146 (0.801, 1.640) 0.45498	1.112 (0.757, 1.632) 0.58809
PDR				
RC (mg/dL)	1.003 (0.996, 1.011) 0.38149	1.002 (0.993, 1.010) 0.71030	1.009 (0.997, 1.022) 0.14995	1.008 (0.995, 1.021) 0.20586
RC (Quartile)				
Q1	Ref.	Ref.	Ref.	Ref.
Q2	1.213 (0.858, 1.714) 0.27367	1.182 (0.828, 1.686) 0.35665	1.219 (0.849, 1.751) 0.28371	1.226 (0.851, 1.767) 0.27444
Q3	1.435 (1.016, 2.025) 0.04023	1.388 (0.972, 1.982) 0.07125	1.559 (1.071, 2.271) 0.02056	1.552 (1.060, 2.273) 0.02384
Q4	1.424 (1.012, 2.004) 0.04270	1.341 (0.938, 1.917) 0.10751	1.709 (1.096, 2.666) 0.01812	1.649 (1.048, 2.593) 0.03047

Crude model: we did not adjust other covariants

Model I: we adjusted age, sex, BMI, SBP, DBP, coronary artery disease, cerebrovascular disease, β-blocker use, ACEI and/or ARB use, diuretic use, calcium channel blocker use

Model II: we adjusted age, sex, BMI, SBP, DBP, coronary artery disease, cerebrovascular disease, β-blocker use, ACEI and/or ARB use, diuretic use, calcium channel blocker use, FPG, HbA1c, eGFR, TG, microalbuminuria

Model III: we adjusted age (smooth), sex, BMI (smooth), SBP (smooth), DBP (smooth), coronary artery disease, cerebrovascular disease, β-blocker use, ACEI and/or ARB use, diuretic use, calcium channel blocker use, FPG (smooth), HbA1c (smooth), eGFR (smooth), TG (smooth), microalbuminuria

*OR* odds ratios, *CI *confidence, *Ref* reference

We also found no significant relationship between RC and PDR in the crude model, the model I, and model II (Table 2). The trend of ORs and 95% CI were also robust irrespective of the type of covariates adjusted (Crude model: OR = 1.003; 95% CI: 0.996-1.011, the model I: OR = 1.002; 95% CI: 0.993-1.010, model II: OR = 1.009; 95% CI: 0.997-1.022).

### Sensitivity analysis

A series of sensitivity analyses were performed to verify our findings’ robustness. We converted RC from a continuous variable to a categorical variable (according to quartile) and then put the categorical-transformed RC back into the model. The results showed that after RC was transformed into a categorical variable, the trend of the effect sizes in different groups was non-equidistant, which indicated that there might be a non-linear relationship between RC and DR or PDR (Table 2).

In addition, we used a GAM to insert the continuity covariate into the equation as a curve. Model III showed this generally remained consistent with the fully adjusted model (OR for DR = 0.999, 95%CI: 0.989-1.010; OR for PDR = 1.008, 95%CI: 0.995-1.021) (Table 2). Besides, we generated an E-value to assess the sensitivity to unmeasured confounding. The E-values of OR for DR and PDR were 1.02 and 1.07, respectively. The E-value was greater than the relative risk of unmeasured confounders and DR or PDR, suggesting unmeasured or unknown confounders had little effect on the relationship between RC and diabetic retinopathy risk.

### The non-linearity addressing by the generalized additive model

**Fig. 3:**
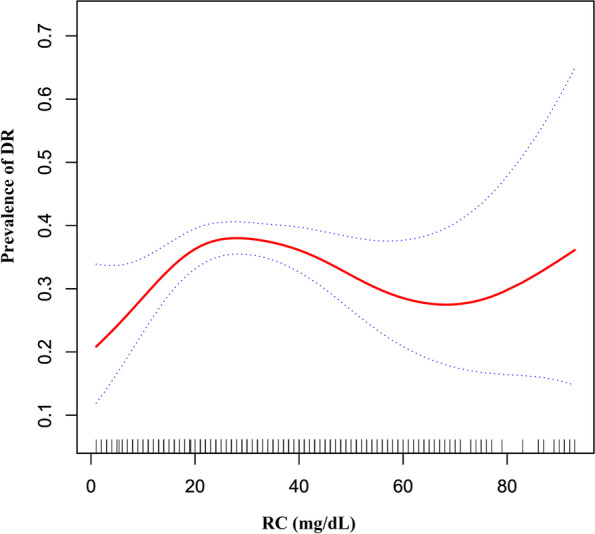
The non-linear relationship between RC and the prevalence of DR. Figure 3. We used a generalized additive model and smooth curve fitting to evaluate the relationship between RC and the risk of DR. The result showed that the relationship between RC and DR was non-linear after adjusting for age, sex, BMI, SBP, DBP, coronary artery disease, cerebrovascular disease, β-blocker use, ACEI and/or ARB use, diuretic use, calcium channel blocker use, FPG, HbA1c, eGFR, TG, and microalbuminuria

Through the GAM and smooth curve fitting, we observed that the association between RC and DR was non-linear (Fig. 3). We fit the data using a standard binary logistic regression model and determine the best fit model using a log-likelihood ratio test (Table 3). In our study, the P for the log-likelihood ratio test was < 0.001. Therefore, we used a two-piecewise logistic regression model to fit the link between the RC and DR. By recursive algorithm, we first obtained the inflection point of RC was 19.0 mg/dL and then calculated the OR and the CI on the left and right of the inflection point. On the left side of the inflection point, the OR and 95%CI were 1.194, (1.070, 1.333), respectively. On the right side of the inflection point, the OR and 95%CI were 0.995, (0.984, 1.006), respectively.

**Table 3:** The result of the two-piecewise logistic regression model for DR

	DR (OR,95%CI)	*P* value
Fitting model by standard logistic regression	1.001 (0.991, 1.011)	0.8555
Fitting model by two-piecewise logistic regression		
Inflection point of RC	19.0 mg/dL	
≤ 13.0 mg/dL	1.194 (1.070, 1.333)	0.0015
> 13.0 mg/dL	0.995 (0.984, 1.006)	0.3668
P for the log-likelihood ratio test	< 0.001	

*OR* Odds ratios, *CI* Confidence, *Ref* Reference, *RC* Remnant cholesterol

We adjusted age, sex, BMI, SBP, DBP, coronary artery disease, cerebrovascular disease, β-blocker use, ACEI and/or ARB use, diuretic use, calcium channel blocker use, FPG, HbA1c, eGFR, TG, microalbuminuria

**Fig. 4:**
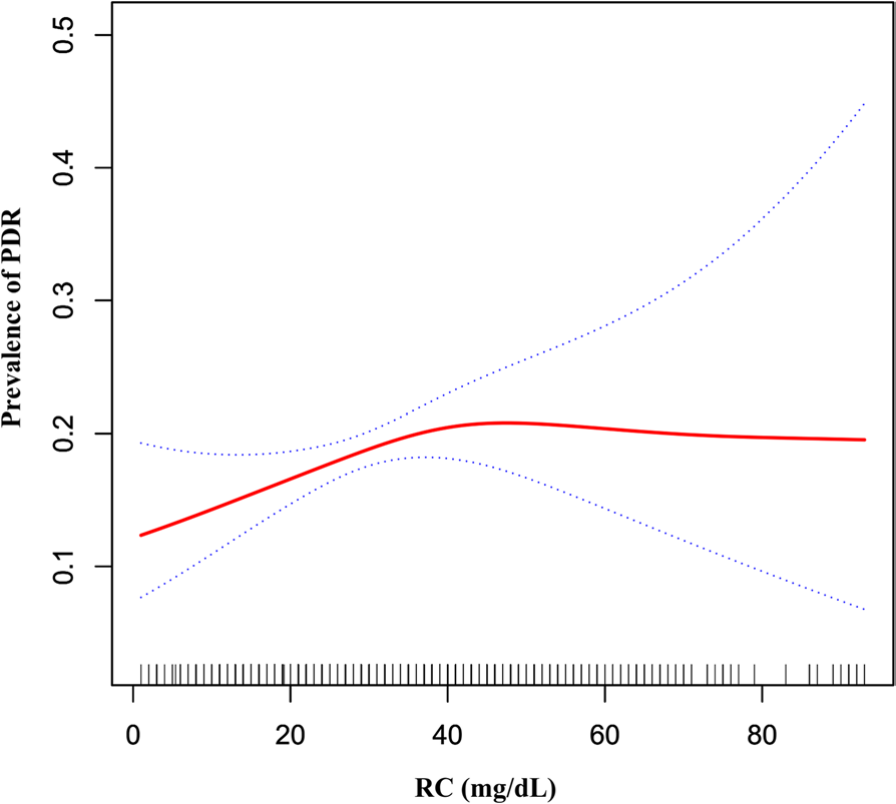
The non-linear relationship between RC and the prevalence of PDR. Figure 4. We used a generalized additive model and smooth curve fitting to evaluate the relationship between RC and the risk of PDR. The result showed that the relationship between RC and DR was also non-linear after adjusting for age, sex, BMI, SBP, DBP, coronary artery disease, cerebrovascular disease, β-blocker use, ACEI and/or ARB use, diuretic use, calcium channel blocker use, FPG, HbA1c, eGFR, TG, and microalbuminuria

We also used the GAM and smooth curve fitting and found that the association between RC and PDR was non-linear (Fig. 4). The inflection point of RC was 39.0 mg/dL. On the left side of the inflection point, the OR and 95%CI were 1.021, (1.004, 1.038), respectively. On the right side of the inflection point, the OR and 95%CI were 0.988, (0.966, 1.012), respectively (Table 4).

**Table 4:** The result of the two-piecewise logistic regression model for PDR

	PDR (OR,95%CI)	P value
Fitting model by standard logistic regression	1.009 (0.997, 1.022)	0.1499
Fitting model by two-piecewise logistic regression		
Inflection point of RC	39.0 mg/dL	
≤39.0 mg/dL	1.021 (1.004, 1.038)	0.0145
> 39.0 mg/dL	0.988 (0.966, 1.012)	0.3340
P for the log-likelihood ratio test	0.028	

*OR* Odds ratios, *CI* Confidence *Ref* Reference, *RC* Remnant cholesterol

We adjusted age, sex, BMI, SBP, DBP, coronary artery disease, cerebrovascular disease, β-blocker use, ACEI and/or ARB use, diuretic use, calcium channel blocker use, FPG, HbA1c, eGFR, TG, microalbuminuria

## Discussion

In a cross-sectional study including 456 Chinese with type 2 diabetes, Shan et al. found that RC levels were positively associated with DR risk [2]. At the same time, the prevalence of DR in their study was 47.59%, whereas it was 35.13% in our study [2]. The patients included in the study by Shan et al. had high levels or proportions of males, HbA1c, FPG, SBP, DBP, TC, and TG, but low levels of HDL-c, as determined by an analysis of their demographic information. Previous studies have reported that these indicators are associated with the risk of developing diabetic retinopathy [3, 21–23]. Consequently, it was accepted that individuals in the current study had a lower prevalence of DR than those in the study by Shan et al.

Furthermore, in contrast to the study by Shan et al .[2], our multivariate logistic regression study did not identify a significant connection between RC and DR or PDR in type 2 diabetic patients. The conflicting results may have been caused by the following: (1) Differences in FPG, blood pressure, blood lipids, and other indicators in the study population (2) Compared with our research, their study did not consider the effect of gender, eGFR, HbA1c, history of coronary artery disease, history of cerebrovascular disease on the relationship between RC and DR when adjusting confounding variables. In addition, these characteristics were addressed in relation to the risk of DR in prior research [21, 22, 24–26]. (3) Differences in sample size may also influence the statistical significance of the association between RC and DR.

Furthermore, to the best of our knowledge, our study observed a non-linear relationship between RC and DR or PDR for the first time. We found a non-linear relation between RC and DR. The inflection point of RC was 13.0 mg/dL. It showed that when RC was below 13.0 mg/dL, a 1 unit increase in the RC levels was associated with a 19.4% increase in adjusted OR for the risk of DR (OR = 1.194, 95%CI: 1.070-1.333). However, when RC > 13.0 mg/dL, a 1 unit increase in the RC level was not associated with the adjusted OR of DR risk (OR = 0.995, 95%CI: 0.984-1.006). Similarly, we also found a non-linear relation between RC and PDR risk, and the inflection point was 39.0 mg/dL. The relationship between RC and PDR on the left and right sides of the inflection point is similar to that between RC and DR. The reason for the difference in the relationship between RC and DR or PDR on both sides of the inflection point might be that other variables also influenced diabetic retinopathy. It could be seen from Table S4 and Table S5 that compared with RC < 13.0 or 39.0 mg/dL, patients with RC ≥ 13.0 or 39.0 mg/dL generally have a higher level or proportion of BMI, BP, FPG, HbA1c, Scr, males, and microalbuminuria. In contrast, participants generally had lower HDL-c and eGFR levels in the RC ≥13.0 or 39.0 mg/dL group. However, the abnormality of the above indicators was closely related to the progress of DR [3, 21–23, 27–29]. When RC was above 13.0 or 39.0 mg/dL, RC had a relatively weak effect on DR progression due to the presence of these DR risk factors. On the contrary, when RC was less than 13.0 or 39.0 mg/dL, the level of the risk factors for DR progressions, such as BMI, BP, HbA1c, FPG, and Scr was lower. The impact on DR was weakened, at this time, the effect of RC was relatively enhanced. Our findings provide an essential rationale for preventing DR and PDR by intervening in the RC level in the clinic. In particular, the RC level should be controlled below 13.0 or 39.0 mg/dL. Because when the RC level is lower than 13.0 or 39.0 mg/dL, the risk of DR or PDR might decrease significantly. The inflection point provides evidence for RC management for the first time.

Our study has some strengths, and we listed them as follows. (1) A strength of our research is that the total sample size was relatively large. (2) Information on covariates is complete and rarely missing. This study explores non-linearity and explains them further. This is a very significant improvement over the previous study. (3) We used multiple imputations to handle missing data in this study. Multiple imputations can maximize statistical power and minimize potential bias caused by covariate information missing. (4) In this study, we ensured the robustness of the results through a series of sensitivity analyses (conversion of target-independent variable form, using a GAM model, calculating E-values to explore the potential for unmeasured confounding). This makes our results more reliable.

Our research has the following shortcomings and needs attention: (1) The design of this study is cross-sectional, so we cannot get the exact causal relationship because of the nature of the cross-sectional design. (2) We only explored the relationship between RC and DR formation and progression in patients with T2DM. Further research is needed on the relationship between the two in patients with type 1 diabetes. (3) As with all observational studies, although known potential confounders such as FPG, TG, and BMI were controlled, there may still be uncontrolled or unmeasured confounders. However, we calculated E-value to quantify the potential impact of unmeasured confounders and found that unmeasured confounders were unlikely to explain the results. (4) As this is a secondary analysis, the raw data did not provide sufficient information on the study population. In the future, we can consider designing our studies or collaborating with other researchers to collect sufficient information on the study population.

## Conclusion

This study demonstrates a non-linear relationship between RC and DR or PDR in patients with T2DM. When the RC level is lower than 13.0 or 39.0 mg/dL, there is a significant positive association with DR and PDR. This result is expected to provide a reference for clinicians to control the RC level. From a treatment perspective, it makes sense to reduce the RC level below the inflection point. Reducing the RC level can significantly reduce DR and PDR risk when the RC level is below the inflection point.

## Supplementary Information

**Additional file 1.**

**Additional file 2.**

## Data Availability

Data can be downloaded from (10.1371/journal.pone.0134718). Under the terms of the Creative Commons Attribution License, which permits unrestricted use, distribution, and reproduction in any medium, provided the original author and source are credited [9].

## Abbreviations

RC: Remnant cholesterol
HDL-c: High-density lipoprotein cholesterol
HbA1c: Hemoglobin A1c
eGFR: Estimated glomerular filtration rate
BMI: Body mass index
Scr: Serum creatinine
FPG: Fasting plasma glucose
LDL-c: Low-density lipoprotein cholesterol
TC: Total cholesterol
TG: Triglyceride
SBP: Systolic blood pressure
DBP: Diastolic blood pressure
PP: Pulse pressure
non-HDL: Non-high-density lipoprotein cholesterol
ACEI: angiotensin converting enzyme inhibitor
ARB: angiotensin II receptor blocker
DR: Diabetic retinopathy
PDR: Proliferative diabetic retinopathy
OR: odds ratio
Ref: Reference
CI: Confidence intervals
T2DM: Type 2 diabetes mellitus
DM: Diabetes mellitus
GAM: Generalized additive model
IDL: Intermediate-density lipoprotein
VLDL: Very-low-density lipoprotein
MDRD: Modification of diet in renal disease
MAR: Missing-at-random
