# Supplementary material for: The non-linear link between remnant cholesterol and diabetic retinopathy: a cross-sectional study in patients with type 2 diabetic mellitus
Source: BMC Endocr Disord. 2022 Dec 21;22:326. doi: 10.1186/s12902-022-01239-5 (PMC9768989; doi:10.1186/s12902-022-01239-5)
Supplement: Supplementary file 1 — Additional file 1. [file 12902_2022_1239_MOESM1_ESM.docx]

**The non-linear link between remnant cholesterol and diabetic retinopathy: a cross-sectional study in patients with type 2 diabetic mellitus**

**Running title:** The association between RC and DR

**Wushan Pan^1^**^#^**, Yong Han^2,3,^**^#^**, Haofei Hu^4,5^*,Yongcheng He^6^***

^1^Department of Nephrology, Kaifeng Central Hospital, Kaifeng 475000, Henan Province, China

^2^Department of Emergency, Shenzhen Second People’s Hospital, Shenzhen 518000, Guangdong Province, China

^3^Department of Emergency, The First Affiliated Hospital of Shenzhen University, Shenzhen 518000, Guangdong Province, China

^4^Department of Nephrology, Shenzhen Second People’s Hospital, Shenzhen 518000, Guangdong Province, China

^5^Department of Nephrology, The First Affiliated Hospital of Shenzhen University, Shenzhen 518000, Guangdong Province, China

^6^Department of Nephrology, Shenzhen Hengsheng Hospital, Shenzhen 518000, Guangdong Province, China

**^#^** Wushan Pan and Yong Han have contributed equally to this work.

*Corresponding author

Haofei Hu,

Department of Nephrology,

Shenzhen Second People’s Hospital,

No.3002 Sungang Road, Futian District,

Shenzhen 518000,

Guangdong Province,

China

Tel:+86-755-83366388

E-mail: huhaofei0319@126.com

Yongcheng He,

Department of Nephrology,

Shenzhen Hengsheng Hospital,

No. 20 Yintian Road, Baoan District,

Shenzhen 518000,

Guangdong Province,

China

E-mail: heyongcheng640815@126.com

## TableS1. The Baseline Characteristics of participants according to diabetic retinopathy

| Variable | Non-DR | Non-PDR | PDR | P-value |
| --- | --- | --- | --- | --- |
| Participants | 1274 | 334 | 356 |  |
| Age (years) | 63.3 ± 11.8 | 66.2 ± 10.4 | 64.8 ± 9.9 | <0.001 |
| BMI (kg/m^2^) | 25.8 ± 3.6 | 25.8 ± 3.5 | 26.1 ± 3.3 | 0.396 |
| SBP (mmHg) | 133.6 ± 18.4 | 134.8 ± 18.5 | 139.4 ± 19.9 | <0.001 |
| DBP (mmHg) | 77.8 ± 11.1 | 77.4 ± 11.6 | 77.9 ± 11.9 | 0.852 |
| PP (mmHg) | 55.8 ± 14.9 | 57.4 ± 15.6 | 61.5 ± 15.5 | <0.001 |
| HbA1c (%) | 7.6 ± 1.7 | 7.6 ± 1.6 | 7.9 ± 1.5 | 0.009 |
| FPG (mg/dL) | 147.6 ± 51.1 | 146.0 ± 45.2 | 151.6 ± 55.0 | 0.296 |
| TC (mg/dL) | 185.3 ± 35.6 | 181.7 ± 34.8 | 183.5 ± 36.9 | 0.229 |
| TG (mg/dL) | 146.6 ± 88.9 | 147.7 ± 85.6 | 144.5 ± 77.7 | 0.884 |
| LDL-c (mg/dL) | 104.7 ± 27.6 | 102.9 ± 28.0 | 103.9 ± 27.1 | 0.540 |
| HDL-c (mg/dL) | 50.2 ± 13.3 | 48.6 ± 13.5 | 48.4 ± 11.9 | 0.026 |
| non-HDL (mg/dL) | 135.2 ± 34.7 | 133.1 ± 32.7 | 135.1 ± 34.9 | 0.626 |
| RC (mg/dL) | 30.5 ± 15.0 | 30.3 ± 14.2 | 31.2 ± 13.5 | 0.667 |
| Scr (mg/mL) | 1.0 ± 0.4 | 1.1 ± 0.4 | 1.2 ± 0.5 | <0.001 |
| eGFR (mL/min/1.73 m^2^) | 70.4 ± 19.4 | 65.6 ± 19.5 | 65.2 ± 20.2 | <0.001 |
| Gender |  |  |  | 0.332 |
| Female | 734 (57.6%) | 196 (58.7%) | 191 (53.7%) |  |
| Male | 540 (42.4%) | 138 (41.3%) | 165 (46.3%) |  |
| Microalbuminuria (%) | 378 (29.7%) | 131 (39.2%) | 174 (48.9% | <0.001 |
| Coronary artery disease (%) | 191 (15.0%) | 70 (21.0%) | 67 (18.8%) | 0.017 |
| Cerebrovascular disease (%) | 48 (3.8%) | 17 (5.1%) | 31 (8.7%) | <0.001 |
| β-blocker use (%) | 284 (22.3%) | 79 (23.7%) | 95 (26.7%) | 0.220 |
| ACEI and/or ARB use (%) | 900 (70.6%) | 248 (74.3%) | 298 (83.7%) | <0.001 |
| Diuretic use (%) | 542 (42.5%) | 155 (46.4%) | 207 (58.1%) | <0.001 |
| Calcium channel blocker use (%) | 438 (34.4%) | 154 (46.1%) | 177 (49.7%) | <0.001 |

Values are n (%) or mean ± SD

Abbreviations. HbA1c, hemoglobin A1c; eGFR, estimated glomerular filtration rate; BMI, body mass index; Scr, serum creatinine; FPG, fasting plasma glucose; HDL-c, high-density lipoprotein cholesterol; LDL-c, low-density lipoprotein cholesterol; TC, total cholesterol; TG, triglyceride; SBP, systolic blood pressure; DBP, diastolic blood pressure; PP, pulse pressure; non-HDL, non- high-density lipoprotein cholesterol; RC, remnant cholesterol; ACEI, angiotensin converting enzyme inhibitor; ARB, angiotensin II receptor blocker; DR, diabetic retinopathy; PDR, proliferative diabetic retinopathy

**Table S2 Prevalence rate of DR and proliferative DR**

| RC group | Participants(n) | DR(n) | Prevalence of DR (95% CI)(%) | PDR(n) | Prevalence of PDR (95% CI) (%) |
| --- | --- | --- | --- | --- | --- |
| Total | 1964 | 690 | 35.13 (33.02-37.25) | 356 | 18.13 (16.42-19.83) |
| Q1 | 438 | 133 | 30.37 (26.04-34.69) | 65 | 14.84 (11.50-18.18) |
| Q2 | 533 | 204 | 38.27 (34.13-42.41) | 93 | 17.45 (14.22-20.68) |
| Q3 | 485 | 177 | 36.49 (32.20-40.79) | 97 | 20.00 (16.43-23.57) |
| Q4 | 508 | 176 | 34.65 (30.49-38.80) | 101 | 19.88 (16.40-23.36) |

RC, remnant cholesterol; DR, diabetic retinopathy; PDR, proliferative diabetic retinopathy

**Table S3. The results of univariate analysis**

| Variable | Statistics | DR [OR (95%CI) P value] | MDR [OR (95%CI) P value] |
| --- | --- | --- | --- |
| Age (years) | 64.103 ± 11.319 | 1.017 (1.009, 1.026) 0.00006 | 1.007 (0.997, 1.018) 0.17283 |
| Gender |  |  |  |
| Female | 1121 (57.077%) | Ref. | Ref. |
| Male | 843 (42.923%) | 1.064 (0.883, 1.283) 0.51402 | 1.185 (0.941, 1.492) 0.14923 |
| BMI(kg/m^2^) | 25.830 ± 3.549 | 1.010 (0.984, 1.037) 0.45629 | 1.022 (0.990, 1.055) 0.17616 |
| SBP(mmHg) | 134.879 ± 18.767 | 1.010 (1.005, 1.015) 0.00006 | 1.015 (1.009, 1.021) <0.00001 |
| DBP(mmHg) | 77.746 ± 11.298 | 0.999 (0.991, 1.007) 0.81784 | 1.001 (0.991, 1.011) 0.80236 |
| PP(mmHg) | 57.132 ± 15.269 | 1.016 (1.010, 1.022) <0.00001 | 1.022 (1.015, 1.030) <0.00001 |
| HbA1c(%) | 7.641 ± 1.627 | 1.069 (1.010, 1.130) 0.02071 | 1.107 (1.036, 1.183) 0.00269 |
| FPG(mg/mL) | 148.046 ± 50.896 | 1.000 (0.999, 1.002) 0.58980 | 1.002 (0.999, 1.004) 0.14169 |
| TC(mg/mL) | 184.388 ± 35.713 | 0.998 (0.995, 1.000) 0.11133 | 0.999 (0.996, 1.002) 0.59722 |
| TG(mg/mL) | 146.394 ± 86.406 | 1.000 (0.999, 1.001) 0.90654 | 1.000 (0.998, 1.001) 0.65499 |
| LDL-c(mg/mL) | 104.237 ± 27.541 | 0.998 (0.995, 1.002) 0.31830 | 0.999 (0.995, 1.004) 0.79299 |
| HDL-c(mg/mL) | 49.578 ± 13.070 | 0.990 (0.983, 0.997) 0.00724 | 0.991 (0.982, 1.000) 0.06194 |
| non-HDL(mg/mL) | 134.810 ± 34.410 | 0.999 (0.996, 1.002) 0.52776 | 1.000 (0.997, 1.004) 0.87178 |
| RC(mg/mL) | 30.573 ± 14.600 | 1.001 (0.995, 1.008) 0.69304 | 1.003 (0.996, 1.011) 0.38149 |
| Scr(mg/mL) | 1.078 ± 0.383 | 1.767 (1.382, 2.261) <0.00001 | 1.705 (1.306, 2.225) 0.00009 |
| eGFR(mL/min/1.73 m^2^) | 68.674 ± 19.669 | 0.987 (0.982, 0.992) <0.00001 | 0.989 (0.983, 0.995) 0.00025 |
| Microalbuminuria |  |  |  |
| No | 1281 (65.224% | Ref. | Ref. |
| Yes | 683 (34.776%) | 1.878 (1.549, 2.276) <0.00001 | 2.064 (1.635, 2.606) <0.00001 |
| Coronary artery disease |  |  |  |
| No | 1636 (83.299%) | Ref. | Ref. |
| Yes | 328 (16.701%) | 1.405 (1.103, 1.790) 0.00595 | 1.196 (0.889, 1.610) 0.23647 |
| Cerebrovascular disease |  |  |  |
| No | 1868 (95.112%) | Ref. | Ref. |
| Yes | 96 (4.888%) | 1.910 (1.266, 2.881) 0.00205 | 2.264 (1.452, 3.531) 0.00031 |
| β-blocker use |  |  |  |
| No | 1506 (76.680%) | Ref. | Ref. |
| Yes | 458 (23.320%) | 1.175 (0.947, 1.460) 0.14352 | 1.248 (0.960, 1.623) 0.09744 |
| ACEI and/or ARB use |  |  |  |
| No | 518 (26.375%) | Ref. | Ref. |
| Yes | 1446 (73.625%) | 1.576 (1.265, 1.963) 0.00005 | 2.059 (1.523, 2.783) <0.00001 |
| Diuretic use |  |  |  |
| No | 1060 (53.971%) | Ref. | Ref. |
| Yes | 904 (46.029%) | 1.491 (1.237, 1.796) 0.00003 | 1.816 (1.439, 2.291) <0.00001 |
| Calcium channel blocker use |  |  |  |
| No | 1195 (60.845%) | Ref. | Ref. |
| Yes | 769 (39.155%) | 1.760 (1.457, 2.126) <0.00001 | 1.697 (1.347, 2.138) <0.00001 |

Values are n(%) or mean±SD

Abbreviations. HbA1c, hemoglobin A1c; eGFR, estimated glomerular filtration rate; BMI, body mass index; Scr, serum creatinine; FPG, fasting plasma glucose; HDL-c, high-density lipoprotein cholesterol; LDL-c, low-density lipoprotein cholesterol; TC, total cholesterol; TG, triglyceride; SBP, systolic blood pressure; DBP, diastolic blood pressure; PP, pulse pressure; non-HDL, non- high-density lipoprotein cholesterol; RC, remnant cholesterol; ACEI, angiotensin converting enzyme inhibitor; ARB, angiotensin II receptor blocker.

## Table S4. The baseline characteristics of participants on both sides of the inflection point according to DR.

| RC | <13 | >=39 | P-value |
| --- | --- | --- | --- |
| Participants | 126 | 1838 |  |
| Age (years) | 62.0 ± 14.0 | 64.2 ± 11.1 | 0.028 |
| BMI (kg/m^2^) | 23.9 ± 2.9 | 26.0 ± 3.6 | <0.001 |
| SBP (mmHg) | 131.4 ± 20.0 | 135.1 ± 18.7 | 0.032 |
| DBP (mmHg) | 75.5 ± 10.9 | 77.9 ± 11.3 | 0.022 |
| PP (mmHg) | 55.9 ± 17.2 | 57.2 ± 15.1 | 0.348 |
| HbA1c (%) | 7.5 ± 1.6 | 7.7 ± 1.6 | 0.332 |
| FPG (mg/mL) | 140.2 ± 56.8 | 148.6 ± 50.4 | 0.074 |
| TC (mg/mL) | 157.8 ± 31.4 | 186.2 ± 35.3 | <0.001 |
| TG (mg/mL) | 73.9 ± 25.8 | 151.4 ± 86.9 | <0.001 |
| LDL-c (mg/mL) | 90.9 ± 27.2 | 105.2 ± 27.3 | <0.001 |
| HDL-c (mg/mL) | 57.8 ± 15.7 | 49.0 ± 12.7 | <0.001 |
| non-HDL (mg/mL) | 100.0 ± 27.0 | 137.2 ± 33.6 | <0.001 |
| RC (mg/mL) | 9.1 ± 2.8 | 32.0 ± 13.9 | <0.001 |
| Scr (mg/mL) | 1.0 ± 0.3 | 1.1 ± 0.4 | 0.277 |
| eGFR (mL/min/1.73 m^2^) | 72.2 ± 18.5 | 68.4 ± 19.7 | 0.038 |
| Gender |  |  | 0.097 |
| Female | 63 (50.0%) | 1058 (57.6%) |  |
| Male | 63 (50.0%) | 780 (42.4%) |  |
| Microalbuminuria (%) | 34 (27.0%) | 649 (35.3%) | 0.058 |
| Coronary artery disease (%) | 19 (15.1%) | 309 (16.8%) | 0.614 |
| Cerebrovascular disease (%) | 2 (1.6%) | 94 (5.1%) | 0.076 |
| β-blocker use (%) | 21 (16.7%) | 437 (23.8%) | 0.068 |
| ACEI and/or ARB use (%) | 74 (58.7%) | 1372 (74.6%) | <0.001 |
| Diuretic use (%) | 50 (39.7%) | 854 (46.5%) | 0.140 |
| Calcium channel blocker use (%) | 34 (27.0%) | 735 (40.0%) | 0.004 |

Values are n (%) or mean ± SD

Abbreviations. HbA1c, hemoglobin A1c; eGFR, estimated glomerular filtration rate; BMI, body mass index; Scr, serum creatinine; FPG, fasting plasma glucose; HDL-c, high-density lipoprotein cholesterol; LDL-c, low-density lipoprotein cholesterol; TC, total cholesterol; TG, triglyceride; SBP, systolic blood pressure; DBP, diastolic blood pressure; PP, pulse pressure; non-HDL, non- high-density lipoprotein cholesterol; RC, remnant cholesterol; ACEI, angiotensin converting enzyme inhibitor; ARB, angiotensin II receptor blocker;

## Table S5. The Baseline Characteristics of participants on both sides of the inflection point according to PDR.

| RC | <39 | >=39 | P-value |
| --- | --- | --- | --- |
| Participants | 1482 | 482 |  |
| Age (years) | 64.2 ± 11.5 | 63.8 ± 10.6 | 0.477 |
| BMI (kg/m^2^) | 25.6 ± 3.6 | 26.5 ± 3.3 | <0.001 |
| SBP (mmHg) | 134.1 ± 18.5 | 137.2 ± 19.4 | 0.001 |
| DBP (mmHg) | 77.1 ± 10.9 | 79.8 ± 12.4 | <0.001 |
| PP (mmHg) | 57.0 ± 15.3 | 57.4 ± 15.2 | 0.625 |
| HbA1c (%) | 7.5 ± 1.6 | 8.0 ± 1.7 | <0.001 |
| FPG (mg/dL) | 143.6 ± 49.0 | 161.6 ± 54.3 | <0.001 |
| TC (mg/dL) | 175.4 ± 29.8 | 212.1 ± 38.0 | <0.001 |
| TG (mg/dL) | 116.3 ± 45.8 | 238.8 ± 112.6 | <0.001 |
| LDL-c (mg/dL) | 100.2 ± 24.8 | 116.7 ± 31.6 | <0.001 |
| HDL-c (mg/dL) | 51.1 ± 13.2 | 44.9 ± 11.4 | <0.001 |
| non-HDL (mg/dL) | 124.3 ± 27.5 | 167.2 ± 33.4 | <0.001 |
| RC (mg/dL) | 24.1 ± 8.1 | 50.5 ± 12.0 | <0.001 |
| Scr (mg/dL) | 1.1 ± 0.4 | 1.1 ± 0.4 | 0.043 |
| eGFR (mL/min/1.73 m^2^) | 69.2 ± 19.3 | 67.0 ± 20.7 | 0.035 |
| Gender |  |  | 0.074 |
| Female | 829 (55.9%) | 292 (60.6%) |  |
| Male | 653 (44.1%) | 190 (39.4%) |  |
| Microalbuminuria (%) | 451 (30.4%) | 232 (48.1%) | <0.001 |
| Coronary artery disease (%) | 239 (16.1%) | 89 (18.5%) | 0.232 |
| Cerebrovascular disease (%) | 62 (4.2%) | 34 (7.1%) | 0.011 |
| β-blocker use (%) | 326 (22.0%) | 132 (27.4%) | 0.015 |
| ACEI and/or ARB use (%) | 1075 (72.5%) | 371 (77.0%) | 0.055 |
| Diuretic use (%) | 666 (44.9%) | 238 (49.4%) | 0.089 |
| Calcium channel blocker use (%) | 548 (37.0%) | 221 (45.9%) | <0.001 |

Values are n (%) or mean ± SD

Abbreviations. HbA1c, hemoglobin A1c; eGFR, estimated glomerular filtration rate; BMI, body mass index; Scr, serum creatinine; FPG, fasting plasma glucose; HDL-c, high-density lipoprotein cholesterol; LDL-c, low-density lipoprotein cholesterol; TC, total cholesterol; TG, triglyceride; SBP, systolic blood pressure; DBP, diastolic blood pressure; PP, pulse pressure; non-HDL, non- high-density lipoprotein cholesterol; RC, remnant cholesterol; ACEI, angiotensin converting enzyme inhibitor; ARB, angiotensin II receptor blocker;

**Figure S1. The RC levels in the three groups of diabetic retinopathy status (non-DR, nonPDR, and PDR).**

Figure S1 indicated that the distribution level of RC in the three groups (non-DR, non-PDR, and PDR). The results indicated that there were no statistically significant differences in the distribution levels of RC in the three groups (P=0.6665).
