# Supplementary figures and images for: The non-linear link between remnant cholesterol and diabetic retinopathy: a cross-sectional study in patients with type 2 diabetic mellitus
Source: BMC Endocr Disord. 2022 Dec 21;22:326. doi: 10.1186/s12902-022-01239-5 (PMC9768989; doi:10.1186/s12902-022-01239-5)

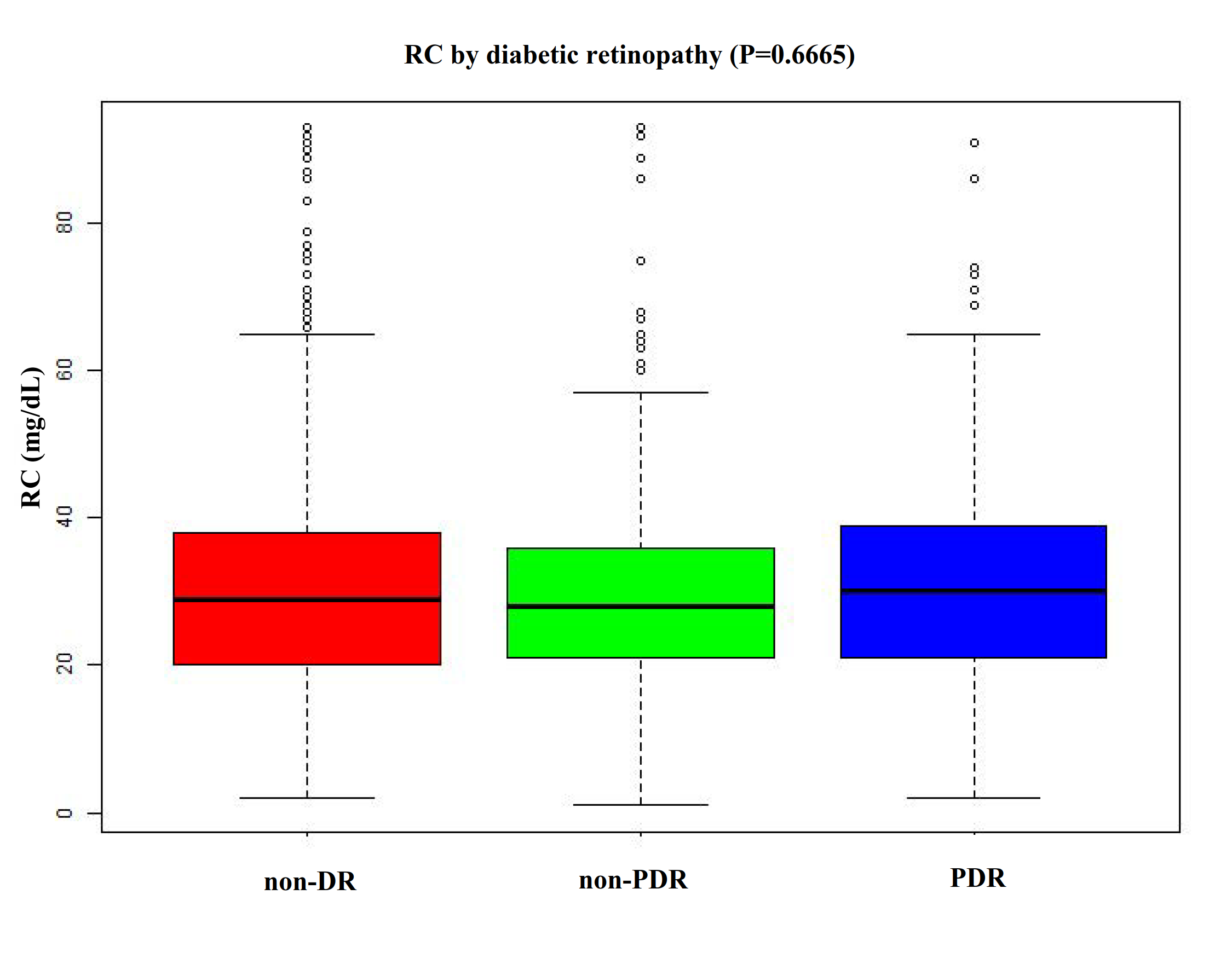

Supplement: Supplementary file 2 — Additional file 2. [file 12902_2022_1239_MOESM2_ESM.tif]
